# Supplementary material for: Ovarian Stimulation in Mice Resulted in Abnormal Placentation through Its Effects on Proliferation and Cytokine Production of Uterine NK Cells
Source: Int J Mol Sci. 2023 Mar 21;24(6):5907. doi: 10.3390/ijms24065907 (PMC10054838; doi:10.3390/ijms24065907)
Supplement: Supplementary file 1 [file ijms-24-05907-s001.zip › ijms-2247003-supplementary.pdf]

Supplementary Material

1 Supplementary Data

Supplementary Table S1 Q-RT-PCR primers sequences for expressed imprinted genes.

**Name**	**Forword（5’→3’）**	**Reverse（5’→3’）**
CXCL10	CAACTGCATCCATATCGATGAC	GATTCCGGATTCAGACATCTCT
CXCL11	GTAACGGCTGCGACAAAGTTGAAG	GAGGCGAGCTTGCTTGGATCTG
CXCL12	TGCATCAGTGACGGTAAACCA	TTCTTCAGCCGTGCAACAATC
CXCL14	GAAGATGGTTATCGTCACCACC	CGTTCCAGGCATTGTACCACT
CCL2	TTAAAAACCTGGATCGGAACCAA	GCATTAGCTTCAGATTTACGGGT
CX3CL1	ACGAAATGCGAAATCATGTGC	CTGTGTCGTCTCCAGGACAA
IL-15	ACATCCATCTCGTGCTACTTGT	GCCTCTGTTTTAGGGAGACCT
IL-24	GAGCCTGCCCAACTTTTTGTG	TGTGTTGAAGAAAGGGCCAGT
PLGF	TCTGCTGGGAACAACTCAACA	GTGAGACACCTCATCAGGGTAT
TGF-β	CTCCCGTGGCTTCTAGTGC	GCCTTAGTTTGGACAGGATCTG
dPRP	TTATGGGTGCATGGATCACTCC	CCCACGTAAGGTCATCATGGAT
GAPDH	GGTGAAGGTCGGTGTGAACG	CTCGCTCCTGGAAGATGGTG

Figure S1 Isolated endometrial stromal cells was determined by immunofluorescence staining

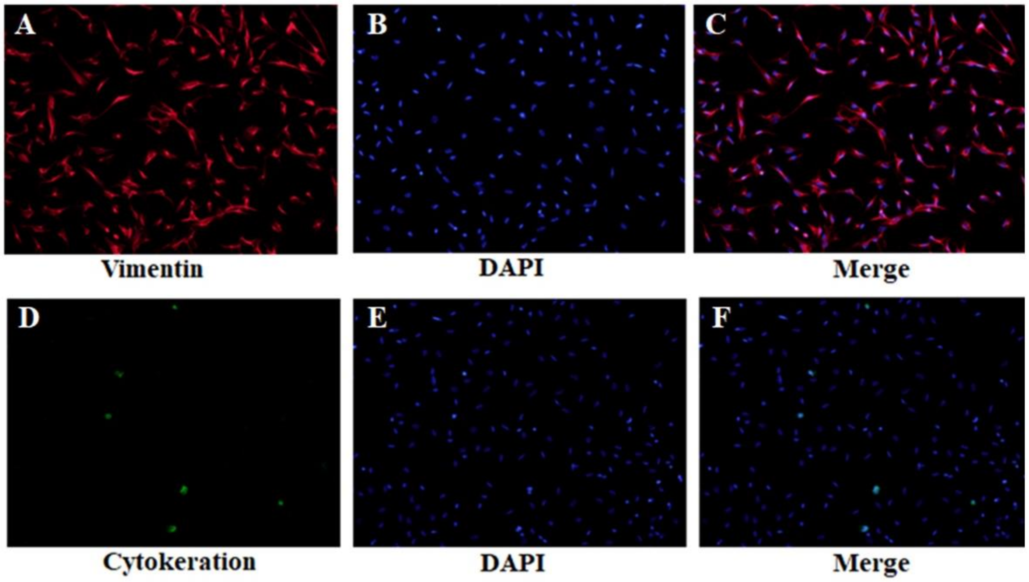

Figure S1. Identification of mouse endometrial stromal cells （100×）

A：Vimentin； B：DAPI；C：Vimentin + DAPI；D：Cytokeration；E：DAPI；F：Cytokeration + DAPI

Figure S2 In vitro decidualization by detecting the expression of dPRP

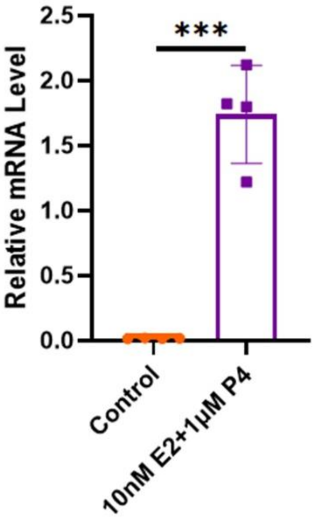

Figure S2. The expression of dPRP before or after decidualization
